# POLICY SERIES: ENSURING SCALE-UP AND SPREAD OF EVIDENCE-BASED PRACTICE: AGE-FRIENDLY HEALTH SYSTEMS IMPLEMENTATION BOOSTERS

**DOI:** 10.1093/geroni/igae098.0476

**Published:** 2024-12-31

**Authors:** Nicholas Schiltz, Heba Aldossary

**Affiliations:** Chair; Co-Chair

## Abstract

This symposium presents a series of studies focused on the implementation of the Age-Friendly Health System (AFHS) 4Ms Framework within the largest convenient care health system in the U.S. These studies highlight the need for innovative “booster” strategies to improve the delivery of the 4Ms—Mobility, Medications, Mentation, and What Matters Most. The first study provides an overview of the implementation and improvement science methods used to facilitate the adoption of the 4Ms. Despite initial efforts, delivery of 4Ms care remained low after one year, highlighting the need for additional booster strategies. The second study introduces the Virtual Clinic, a serious video game designed to improve provider competency and confidence in delivering 4Ms care. The game simulates realistic older adult patient visits, providing valuable practice for providers. The third study presents the Reflection and Action strategy, a frontline feedback-driven approach aimed at enhancing both competency and confidence among providers in delivering the 4Ms. The strategy leverages the well-established “Plan, Do, Study, Act” model for improvement. The fourth study discusses the Age-Friendly Health Systems Questionnaire (AFHS-Q), a novel approach developed by frontline providers that enhances efficiency by having patients complete eight 4Ms-related questions while awaiting their medical visit. The final study introduces the Age-Friendly Health Systems Advocacy Tip Sheet, a tool designed to empower older adults and caregivers to advocate for Age-Friendly care. Collectively, these studies emphasize the importance of innovative booster strategies, frontline feedback, and patient empowerment in enhancing the delivery of age-friendly care.

